# The Care of Cutaneous Diseases of the Elephant in Captivity

**Published:** 1882-10

**Authors:** Max Schmidt

**Affiliations:** Director of the Frankfort Zoological Garden


### ART. IV.—THE CARE OF CUTANEOUS DISEASES OF THE ELEPHANT IN CAPTIVITY.

BY DR. MAX SCHMIDT.
(Director of the Frankfort Zoological Garden.)

Abcesses are often formed under the hard epidermis plates in the skin of the elephant. They are generally small, ranging in size from that of a pea to that of a hazelnut. Being covered by the fixed skin, they cannot readily find an opening on the surface. They are usually discovered accidentally by the painful movements of the animal while it is being cleaned whenever the sore spots are touched.

In several instances elephants have been known to carry, by means of their trunks, the hands of their keepers to the painful place. These abcesses are principally caused by the penetration into the skin of filth and foreign matter, such as grains of sand, slivers of wood, etc. The treatment consists simply in thinning down the epidermis until the small pus sac is opened.

Sometimes it is necessary to assist the emptying of the abcess by a slight pressure of the hands from both sides. A cure results without further treatment.

The already thick skin of the elephant frequently gets remarkably thick in single spots, or on a large part of the body appearing like layers laid one upon the other, and covered over with crusts of dust and dirt. This is caused by the neglect of bathing, rubbing and scouring the skin of the animals in captivity, whereby the old epidermis layers are loosened and removed.

As a rule, these crusts are most noticeable in elephants exposed by travelling menageries to dusty roads and housed in filthy stables, whose skin is not only not cared for, but is rather injured by the rubbing on of fat, oil and coloring matter to make them look darker. If left alone these crusts become dry and hard, pressing the skin underneath by each movement or crack, thereby forming very painful chaps.

Collections of skin tallow are often formed under these, which have the appearance of pus formations, irritating the skin by pressure and straining, so that abscesses and tumors may readily be the result. To prevent the occurrence of the conditions above described it is necessary to take special care of the skin of the elephant. If the animals are received young and directly imported, all that is necessary is to keep the skin clean by rubbing it daily with a hard brush, and, in favorable weather, to wash them with water, or, if possible, to let them bathe. Thus the skin will be kept smooth without artificial aids. The greater number of elephants do not arrive at the zoological gardens until after they have been with travelling menageries some time, and their skin is found to be in a filthy condition. It will be necessary, then, to wash the animal thoroughly, using green soap and warm water when the epidermis is greasy. After the washing, and whilst the skin is still soft, the thick dirt and epidermis crusts should be carefully removed by rubbing them with a piece of pumice stone or scraping them with a small iron scraper, until the normal thickness of the skin has been again restored. This takes much time, and must be done carefully, so that the animal, which often becomes impatient, be not injured or provoked to resistance. If oil be not applied after this treatment, the epidermis will become dry and brittle, and chaps will form. To retain the necessary softness it should be applied the day after the skin has been washed. Oil alone should be used for this purpose, applying it with a cloth rather than with a brush, and put on as thin as possible. The oil should be used very seldom, and never except after the skin has been previously cleaned. Dirt accumulating on the oiled skin should be daily removed. Glycerine has lately been tried instead of oil, with the best results, the old crusts loosening more easily, new ones not forming, whilst the skin becomes remarkably soft. The washing must be done only during mild weather, as it might lead to colds, and should never be attempted during the winter months.

